# DNA metabarcoding reveals high relative abundance of trunk disease fungi in grapevines from Marlborough, New Zealand

**DOI:** 10.1186/s12866-022-02520-2

**Published:** 2022-05-10

**Authors:** Bhanupratap R. Vanga, Preeti Panda, Anish S. Shah, Sarah Thompson, Rebecca H. Woolley, Hayley J. Ridgway, Dion C. Mundy, Simon Bulman

**Affiliations:** 1 Canterbury Agriculture and Science Centre, The New Zealand Institute for Plant and Food Research Limited, Gerald St, Lincoln, 7608 New Zealand; 2 Department of Pest Management and Conservation, Faculty of Agriculture and Life Sciences, Lincoln University, P O Box 84, Lincoln, 7647 New Zealand; 3 Marlborough Wine Research Centre, The New Zealand Institute for Plant and Food Research Limited, PO Box 845, Blenheim, New Zealand; 4 Better Border Biosecurity (B3), Lincoln, 7608 New Zealand

**Keywords:** DNA metabarcoding, Grapevine trunk disease, Fungi, Pathogen identification, Specific PCR

## Abstract

Grapevine trunk diseases (GTDs) are a threat to grape production worldwide, with a diverse collection of fungal species implicated in disease onset. Due to the long-term and complex nature of GTDs, simultaneous detection of multiple microbial species can enhance understanding of disease development. We used DNA metabarcoding of ribosomal internal transcribed spacer 1 (ITS1) sequences, supported by specific PCR and microbial isolation, to establish the presence of trunk pathogens across 11 vineyards (11–26 years old) over three years in Marlborough, the largest wine producing region in New Zealand. Using a reference database of trunk pathogen sequences, species previously associated with GTD, such as *Cadophora luteo-olivacea*, *Diplodia seriata, Diplodia mutila*, *Neofusicoccum australe,* and *Seimatosporium vitis*, were identified as highly represented across the vineyard region. The well-known pathogens *Phaeomoniella chlamydospora* and *Eutypa lata* had especially high relative abundance across the dataset, with *P. chlamydospora* reads present between 22 and 84% (average 52%) across the vineyards. Screening of sequences against broader, publicly available databases revealed further fungal species within families and orders known to contain pathogens, many of which appeared to be endemic to New Zealand. The presence of several wood-rotting basidiomycetes (mostly Hymenochaetales) was detected for the first time in the Marlborough vineyard region, notably, the native *Inonotus nothofagii* which was present at 1–2% relative abundance in two vineyards.

**Supplementary Information:**

The online version contains supplementary material available at 10.1186/s12866-022-02520-2.

## Introduction

Grapevine trunk diseases (GTDs) infect trunk vascular tissue in grapevines (*Vitis vinifera*). Symptoms include cankers that often appear as V-shaped lesions in cross-sections of perennial tissue. Rotting of trunk tissues is associated with shoot stunting, foliar chlorosis, reduced berry yield, and, eventually, vine death [1].

GTDs are increasing in incidence and threaten the productivity of vineyards worldwide [2]. Vineyards reach a peak of trunk disease incidence at 15–20 years old. Despite the younger average age of vineyards (18 years; W. Kerner pers. comm.), GTDs are already important in New Zealand, with approximately 9% of vines displaying disease symptoms in Marlborough and Hawke’s Bay [3]. The predominance of the susceptible Sauvignon blanc variety raises the possibility that New Zealand vineyards are predisposed to future disease problems [4].

Research to define the role of pathogenic fungi has focused on key taxa associated with three important categories of trunk disease; *Phaeomoniella chlamydospora* and *Phaeoacremonium* spp. with esca disease, Botryosphaeriaceae spp. with botryosphaeria dieback and *Eutypa lata*/Diatrypaceae spp. with eutypa dieback. However, culture-dependent studies have associated as many as 133 fungal species from 34 genera with GTD – albeit not always with fulfilment of Koch’s postulates [2]. Interactions between multiple fungal species likely determine the outcome of disease [5]. Adding to this complexity, GTD symptoms develop over a period of years, with many fungi being present as latent infections prior to disease expression. This lengthy development makes linkage of disease symptoms to specific fungi especially difficult. The capability of many fungi to cause vascular streaking has been established in pathology assays, however, symptoms may be due to factors other than infection, and replicating field infections over a longer duration is difficult to achieve [6].

New Zealand is distant from other grape-growing countries, with imports of plant material and new grapevine pathogens, severely curtailed. Geographical separation has shaped existing grapevine microbiology; GTD symptoms observed in New Zealand do not encompass all disease forms recorded in older wine-growing regions of the world. In particular, symptoms of the potentially devastating esca disease complex, including foliar interveinal red or white stripes, superficial black fruit spots, or sudden plant wilting have not been reported in New Zealand [4]. The absence of esca may be related to the cool climate and near-ubiquitous use of irrigation, or to the complex of fungi present [4]. *Phaeomoniella chlamydospora*, which is implicated in esca onset, is found in New Zealand vineyards, but wood-rotting hymenochaetes, which are associated with esca white rot [7], have not been detected.

Understanding the progression of GTD is complicated by limitations in available techniques. Culture-dependent methods are constrained by sample throughput, over-representation of fast-growing fungi and difficulties in culturing some species. Quantitative polymerase chain reaction (qPCR) assays [8] provide information on individual species. Specific and scalable diagnostics are required to better understand the temporal changes in microbiology of trunk disease. Morales-Cruz et al. (2018) [9] have initially explored the use of metabarcoding from grapevine trunks, showing that the bulk of GTD fungal taxa could be detected, depending on primer selection.

The microbiology of GTD in New Zealand has been studied using culture-dependent methods [10], but these works have been of insufficient scope to develop a comprehensive picture of trunk microbiology in any region. Many gaps in knowledge remain by comparison to wine-producing regions elsewhere. In this study, we used DNA metabarcoding as a tool to detect multiple fungi in the trunks of Marlborough grapevines. The TrunkDiseaseID database (trunkdiseaseid.org/) [11] was utilised to identify pathogens within the fungal ITS metabarcoding data. Our main goal was to develop a comprehensive register of GTD fungi across vineyards in Marlborough, the largest wine-producing region of New Zealand. Fungal culturing and specific PCR were used to support taxonomic identifications.

## Materials & methods

### Sampling

Grapevine samples were collected from eleven vineyards in Marlborough, which contained either Sauvignon blanc or Pinot noir varieties. In each vineyard, nine sampling loci were selected at random. Five adjacent vines on each row were sampled at each locus. Sampling was repeated in the summers of 2016, 2017 and 2018. One vineyard block was replanted after the 2016 season, and was replaced with a new block in 2017.

Trunk samples were taken above the graft union but below the crown, approximately 80 cm from the ground. Wood tissue was collected using sterilised drill bits [12], snap-frozen in the field and stored at -80 °C until DNA extraction. Holes were sealed with wood filler after sampling. Samples in each subsequent year were collected at least 5 cm away from the previous drill hole.

### Disease assessment

All vines in each locus were visually assessed in January 2017 for dieback, wood cankers and foliar symptoms consistent with trunk disease. Dieback symptoms consisted of at least two dead spurs or canes on one side of a vine. Missing vines were noted but were not counted as symptoms of trunk disease, as it was impossible to determine the cause of their loss.

### DNA metabarcoding

DNA was extracted from individual wood tissue samples using a CTAB method [12]. Apical segments from potato (*Solanum tuberosum*) tissue culture plantlets were included as DNA extraction controls. DNA concentration was estimated by spectrophotometry (NanoDrop, ThermoFisher).

For DNA metabarcoding, composite DNA samples were created by combining equal volumes of DNA from the five vines at each locus. DNA was amplified with Illumina adaptor tagged NSI1a and 58A2R primers, which amplify the fungal ribosomal ITS1 spacer region, spanning from the 18S to the 5.8S ribosomal genes [12]. PCR amplifications with KAPA3G Plant PCR reagents (Sigma-Aldrich) were performed in duplicate. PCR amplicons were purified with the AMPure XP PCR purification system (Agencourt) and quantified using a NanoDrop ND-1000 spectrophotometer (NanoDrop Technologies). Purified PCR amplicons, adjusted to 10 ng/µL, were indexed and Illumina MiSeq pair-end (300 bp PE) sequenced by the New Zealand Genomics Limited (Massey University).

Positive control amplifications were carried out on purified genomic DNA from *Clonostachys rosea*, *Chaetomium* sp., *E. lata* and *Diplodia seriata* isolates. No-template negative controls (NTC) were included during all PCR amplifications.

### Bioinformatic processing

Reads were processed using a USEARCH v11 pipeline [13]. Briefly, paired-end reads were quality trimmed to Q15 and sequences with a maximum expected error rate of 1.0 were discarded. The quality-filtered sequences were merged, de-replicated, sorted by decreasing abundance and singletons removed, prior to clustering into operational taxonomic units (OTUs) at 97% sequence similarity using UPARSE [14]. Chimeric sequences were identified and removed using the de novo implementation of UCHIME [15]. Sequences were mapped back to OTUs using the “usearch_global” algorithm. Raw data are deposited in publicly available National Centre for Biotechnology Information Sequence Read Archive under BioProject accession number PRJNA658210.

### Taxonomic assignment and pathogen identification

For taxonomic identification, OTU sequences were compared against the NCBI nucleotide (‘nt’) and UNITE [16] databases. Taxonomic assignments were inferred using BLASTn 2.5.0 + [17] with the ‘-e’ parameter set to 1e-10 and ‘-max_target_seqs’ and ‘-max_hsps’ parameters set to 3 and 1, respectively. Because our amplified sequences contained more of the conserved ribosomal 18S gene than most database reference sequences, we extracted the ITS regions of OTUs with ITSx [18]. Species-level matches were accepted where the top alignment covered ≥ 85% of the query sequence length, with a minimum sequence identity of 99%. Genus‐level was assigned at ≥ 97% similarity, family-level at 90% and phylum‐level at ≥ 80%. OTUs with ≥ 85% query coverage but blast hits < 80% similarity against the databases were classified as ‘unassigned’. OTUs with very similar matches to multiple reference sequences (percentage sway 0.2%) were denoted as ‘unclassified’.

For diagnosing trunk pathogens, species-level taxonomic identifications were made using BLASTn against a curated reference database of DNA sequences from the TrunkDiseaseID tool (trunkdiseaseid.org) [11]. To identify candidate pathogens beyond those represented in the TrunkDiseaseID tool, we examined the UNITE assignments for known pathogen groups by filtering at higher taxonomic levels (e.g. by examining all assignments within family Diatrypaceae). A final layer of pathogen screening was performed by analysing sequences using the online FUNguild tool [19]. All matches to pathogen sequences were manually re-checked by BLASTn against the NCBI nt database. Presence/absence information for species in New Zealand was obtained from the New Zealand Organisms Register (www.nzor.org.nz).

### Statistical analysis

Statistical analyses were performed within MicrobiomeAnalyst v1.0 [20]. The dataset was rarefied to a minimum library size (resulting in exclusion of 13 samples), followed by normalization using total sum scaling (count per million normalization). Sequencing depth was characterized by Good’s coverage. Alpha diversity measures were assessed using the Shannon index and ANOVA (analysis of variation) methods, implemented using the Phyloseq package [21].

### Specific PCR

Putatively species-specific primers were designed in the ITS1 spacer region of OTU sequences using Primer3 [22] in Geneious [23]. For PCR reactions, specific primers were paired with conserved primers in the ribosomal 18S or 28S genes. For *Phaeoacremonium* PCR we used the primers Pm1F/Pm2R [24] and Lt347-F/Lt347-R [25] for *Lasiodiplodia theobromae*. PCR amplicons were purified and Sanger sequenced (Macrogen Korea) with the PCR primers.

### Fungal isolation from selected vines

For fungal isolation, new wood samples were drilled, as for the DNA extraction, from 40 vines at eight loci across four vineyards. Samples were collected in August 2017 when vines were in winter dormancy. Samples were transported to the laboratory in chilled containers, then plated within 48 h on Malt Extract Agar (MEA) or Potato Dextrose Agar (PDA) supplemented with streptomycin 200 µg/mL. Fungi growing from wood chips were sub-cultured until pure cultures were obtained. DNA was extracted from fungal mycelium using a CTAB protocol [10]. ITS DNA fragments were amplified and sequenced from isolates using ITS5 and ITS26 primers. Electropherograms were edited using Geneious 10.2.5 (http://www.geneious.com/). Matches were generated with BLASTn against the NCBI nt database. The resulting hits were sorted by *e*-value and a putative taxonomy assigned to the isolates at 99% sequence identity.

## Results

### Summary of dataset

A total of 12,657,371 raw reads were generated from 297 samples, of which 12,083,507 were retained after sequence quality control. Following clustering at 97% identity, filtering of chimeras, and removal of samples with < 1500 reads, a total of 1250 OTUs were taxonomically identified, representing 10,928,479 (86.3%) of the total reads. The reads per sample ranged from 1673 to 168,003 with an average of 36,796. After rarefying data to minimum library size, 284 samples remained. Good’s coverage for each sample ranged between 97.6 and 99.5%, implying that the current measurement depth was sufficient to estimate the fungal diversity.

Sequence reads from positive DNA extraction control (*E. lata, D. seriata, Clonostachys rosea and Chaetomium* sp.), amplifications consisted almost exclusively of the target fungal taxa (~ 99%).

Four NTC samples and three DNA extraction controls yielded very low number of sequence reads; 4 (year 1), 7 (year 3), 79 and 325 (year 2) read counts with no major pathogens identified.

### Fungal abundance and diversity

**Fig. 1:**
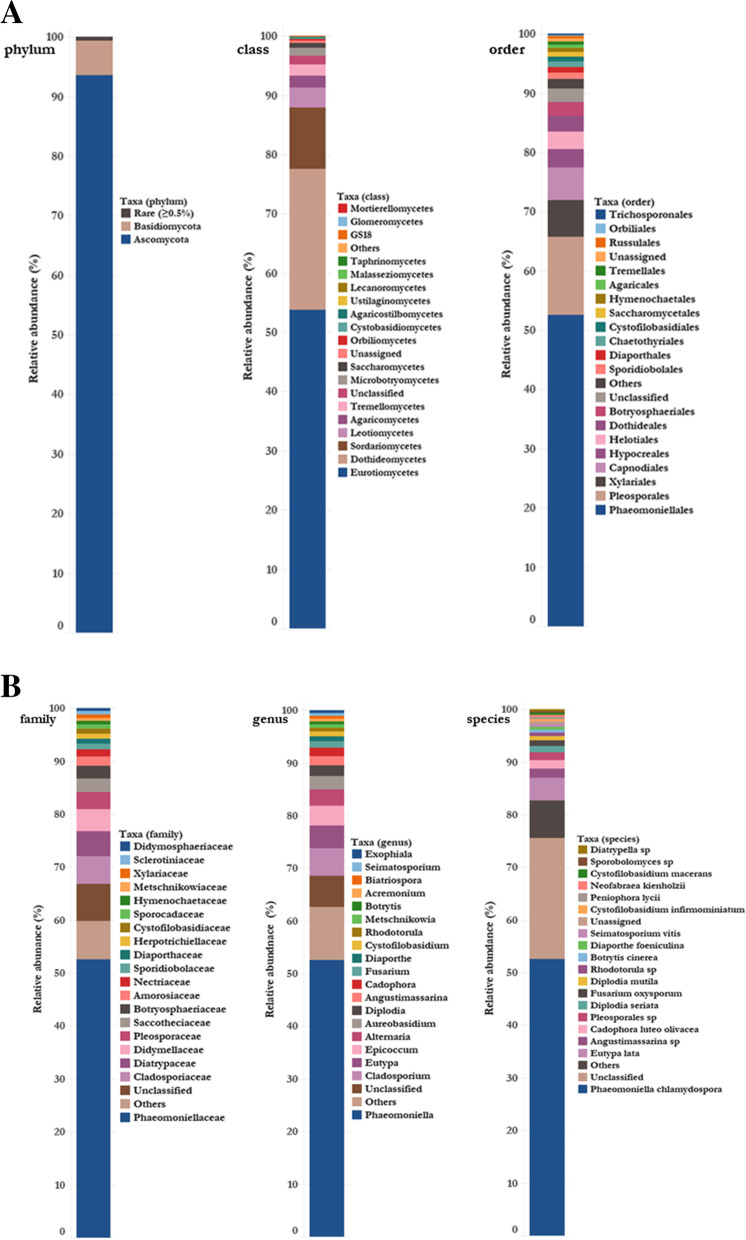
**A** Relative abundance of fungal taxa shown at phylum, class, and order level. **B** Relative abundance of fungal taxa shown at family, genus and species level. ‘Others’ were those taxa with less than 10 read counts

Overall, the fungal distribution spanned 4 phyla (Ascomycota 93.6% and Basidiomycota 5.8%), 19 classes, 52 orders, (Fig. 1A) 106 families, 161 genera and 163 species (excludes ‘unclassified’ and ‘unassigned’ taxa) (Fig. 1B). The genera with the highest relative abundance were *Phaeomoniella* (52.5%), *Cladosporium* (5.2%), *Eutypa* (4.3%), *Epicoccum* (3.7%), *Alternaria* (3.1%), *Aureobasidium* (2.6%), *Diplodia* (2%), *Angustimassarina* (1.7%), *Cadophora* (1.6%), and *Fusarium* (1.3%), together comprising 78% of the total sequences.

**Fig. 2:**
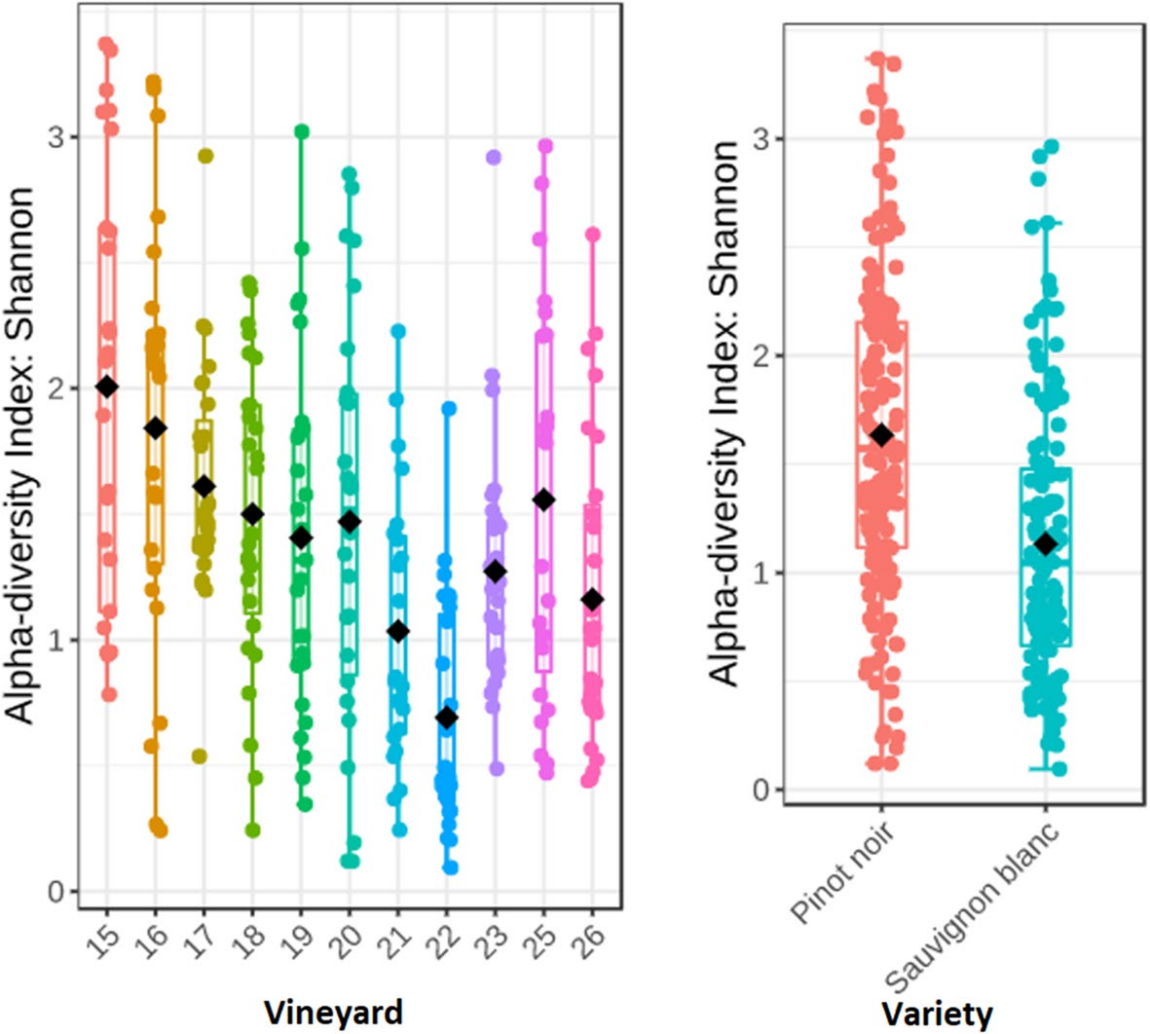
Shannon alpha diversity index box plots for vineyards (*p*-value: 4.56e-11) (left) and variety (right) (*p*-value: 3.94e-09). Vineyards 21, 22, 23, 25 and 26 contained Sauvignon blanc vines while Pinot noir grapevines were grown in the other vineyards

Statistically significant differences in Shannon diversity were observed between vineyards (*p*-value: 4.56e-11) and grape varieties (*p*-value: 3.94e-09) (Fig. 2). Sauvignon blanc vines had a greater alpha diversity than Pinot noir vines. On average, Sauvignon blanc vines had higher relative abundance of *Phaeomoniella*, and *Eutypa*. *Chondrostereum*, *Exophiala*, *Neofabraea* and *Botrytis* were exclusively observed in Pinot noir vines (Supplementary Fig. 1).

### Identifying pathogens

Comparison against the TrunkDiseaseID database showed that 26 of the OTUs matched a reference sequence at > 99% similarity across ≥ 85% coverage (Table 1). Many of the identified taxa had high relative abundance in the dataset, including *P. chlamydospora*, *E. lata*, *D. seriata* and *Cadophora luteo-olivacea*. The greatest number of OTU sequences (eight) with TrunkDiseaseID matches corresponding to Botryosphaeriaceae.

**Table 1:** Known and putative pathogen taxa. A manually curated list of potential trunk pathogen species (Fungal ID) detected by metabarcoding from Marlborough grapevines. Top matching species > 99% similarity are listed; OTUs identification to genera only is based on 97–99% identity or matches to unnamed species. RA % = relative abundance of OTUs across the entire metabarcoding dataset. TD_ID = species identified (Y) by screening against the TrunkdiseaseID reference database. SpPCR = detected by specific PCR. P/A = presence (P) or absence (A) of species in New Zealand from New Zealand Organism Register or based on top matches to unnamed species isolated in New Zealand. U = undetermined, A* = *Cadophora malorum* is listed as unknown in New Zealand by NZOR. — = not done

OTU	Fungal ID	reads	RA%	TD_ID	SpPCR	P/A
OTU_1	*Phaeomoniella chlamydospora*	6,477,112	59.3	Y	—	P
OTU_3	*Eutypa lata*	511,637	4.7	Y	—	P
OTU_14	*Cadophora luteo-olivacea*	168,751	1.5	Y	—	P
OTU_12	*Diplodia seriata*	103,793	0.9	Y	—	P
OTU_23	*Peniophora lycii*	90,568	0.8	—	—	P
OTU_214	*Diplodia mutila*	85,261	0.8	Y	—	P
OTU_15	*Diaporthe foeniculina*	84,667	0.8	—	—	P
OTU_18	*Chondrostereum purpureum*	64,501	0.6	—	Y	P
OTU_16	*Inonotus nothofagi*	40,233	0.4	—	Y	P
OTU_28	*Seimatosporium vitis*	34,123	0.3	—	—	A
OTU_29	*Neofusicoccum australe*	34,109	0.3	Y	—	P
OTU_27	*Diaporthe viticola*	32,370	0.3	—	—	P
OTU_76	Hymenochaetaceae sp.	24,116	0.2	—	Y	U
OTU_648	*Sporocadus rosigena*	19,826	0.2	—	—	P
OTU_1692	Hymenochaetaceae sp.	19,689	0.2	—	—	P
OTU_46	*Diatrypella* sp.	18,399	0.2	Y	Y	P
OTU_59	*Diaporthe neoviticola*	14,124	0.1	—	—	P
OTU_1227	*Diatrypella* sp.	9950	0.1	—	Y	P
OTU_98	*Neofusicoccum parvum*	9194	0.1	Y	—	P
OTU_77	*Phaeomoniella niveniae*	7017	0.1	—	Y	A
OTU_80	*Phaeoacremonium* sp.	6696	0.1		Y	P
OTU_54	Hymenochaetaceae sp.	3946	0.0	—	Y	U
OTU_141	*Pestalotiopsis disseminata*	2806	0.0	Y	—	P
OTU_34	Hymenochaetaceae sp.	2009	0.0	—	—	P
OTU_152	*Morinia acacia*	1425	0.0	—	—	P
OTU_199	*Truncatella angustata*	773	0.0	Y	—	P
OTU_1754	*Phaeoacremonium pseudopanacis*	709	0.0	Y	Y	P
OTU_212	*Cadophora malorum*	646	0.0	—	—	A*
OTU_204	*Resinicium bicolor*	549	0.0	—	—	P
OTU_269	*Diplodia* sp.	352	0.0	Y	—	P
OTU_264	*Phaeoacremonium fraxinopennsylvanicum*	342	0.0	Y	Y	P
OTU_324	*Cryptovalsa ampelina*	201	0.0	Y	—	A
OTU_449	Hymenochaetaceae sp.	196	0.0	—	—	U
OTU_625	*Immersidiscosia eucalypti*	119	0.0	—	—	A
OTU_535	*Schizophyllum commune*	117	0.0	Y	—	P
OTU_474	*Bartalinia robillardoides*	116	0.0	—	—	A

**Fig. 3:**
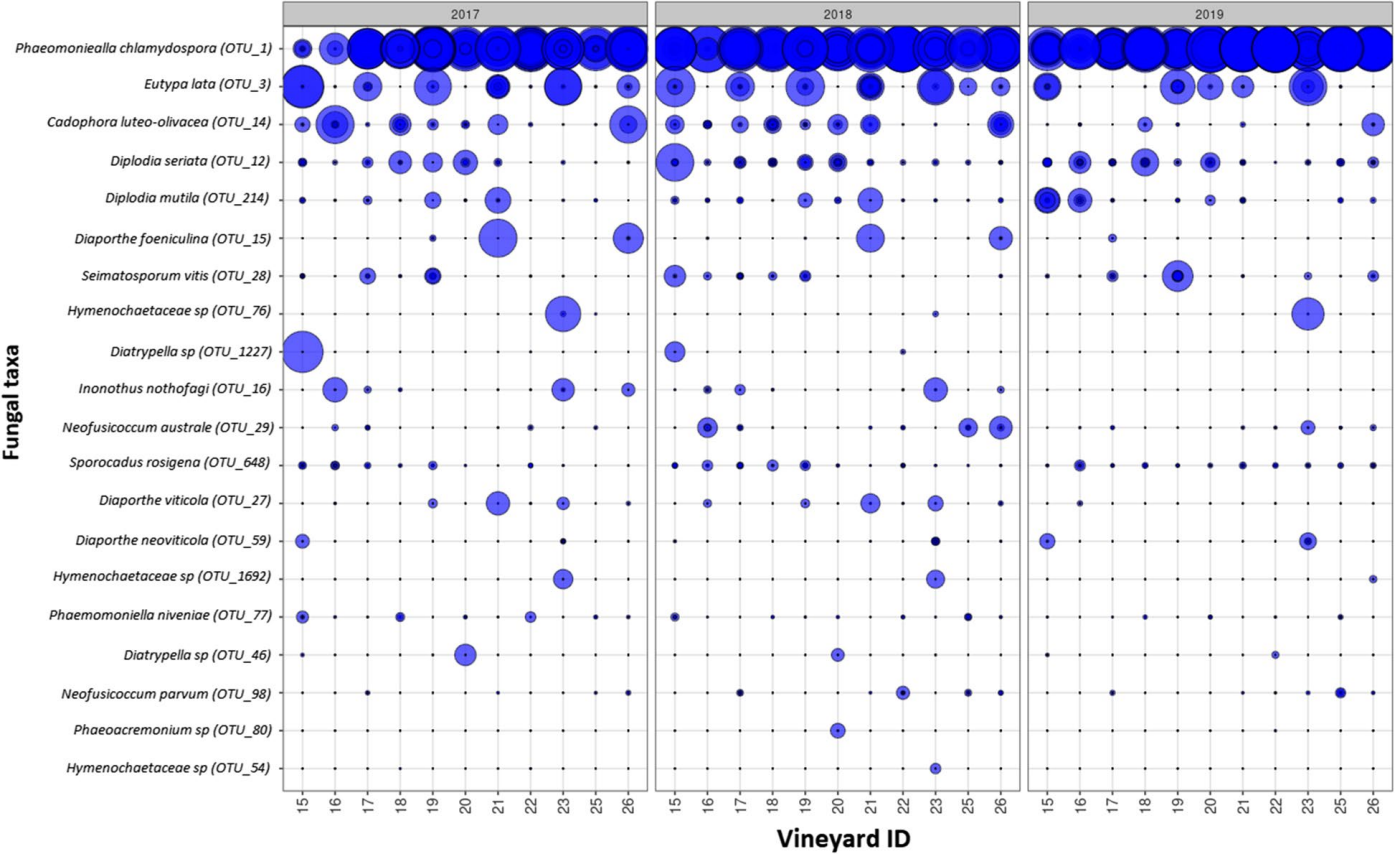
Pathogen diversity in Marlborough grapevines. Bubble plot showing the relative abundance of OTUs for fungal pathogen species across the 11 vineyards in each year from 2017–2019. Bubble size corresponds to the relative abundance of the OTUs

Manual examination of taxonomic identities generated from UNITE database comparisons, revealed 62 OTUs within higher level pathogen taxa, of which 25 were considered “rare” (< 100 reads in total) and excluded from presentation (Table 1). The distribution of the 20 most abundant pathogen taxa across the entire dataset is shown in Fig. 3.

#### Order Botryosphaeriales

Eight OTUs assigned to family Botryosphaeriaceae, made up 2.35% of total reads. Of these reads, *D. seriata* (53.91%), *Diplodia mutila* (30.03%), *Neofusicoccum australe* (11.92%) and *Neofusicoccum parvum* (3.85%) were the most abundant Botryosphaeriaceae species (Table 1). A rare OTU present in two samples matched *L. theobromae*.

#### Order Hymenochaetales

Of total reads, 0.69% across 21 OTUs corresponded to order Hymenochaetales (Table 1). *Inonotus nothofagii* (45%) had the highest relative abundance followed by a putative *Fomitiporella* species and a sequence with closest similarity to *Fomitipora australiensis*. A number of rare OTU sequences corresponded to *Hydnoporia olivacea*, *Tubulicrinis subulatus* and *Hymenochaete porioides*, species not known to be present in New Zealand. Two abundant OTUs, initially classified only as basiodiomycetes, corresponded to a Hymenochaetales species isolated from a New Zealand grapevine [10] and to a putative *Fomitiporia* species.

#### Orders Diaporthales and Togniniales

Of all reads, 0.95% were classified as Diaporthales, of which 64.2% belonged to *Diaporthe foeniculina*, 24.5% as *Diaporthe viticola* and 10.7% *Diaporthe neoviticola*. Rare Diaporthales corresponded predominantly to *Diaporthe* and *Cytospora* (family Valsaceae) species (Table 1).

Only 0.04% of all reads in five OTUs were from Togniniales, consistent with other studies showing *Phaeoacremonium* species present at 1.9–2.6% of fungi in mature vines [26]. All matches were to *Phaeoacremonium fraxinopennyslvanicum*, *P. griseo-olivaceum* and *P. pseudopanacis*, species which have previously been detected in New Zealand [10], although the DNA sequence of the most abundant *Phaeoacremonium* sp. was an imperfect match for *P. pseudopanacis*.

#### Family Phaeomoniellaceae

Of the total reads, 52.6% belonged to the Phaeomoniellaceae family of which *P. chlamydospora* (99.8%) constituted the vast majority. The remaining reads (0.2%) were primarily from *P. niveniae*.

#### Family Diatrypaceae

Of total reads, 4.7% were assigned to Diatrypaceae. *Eutypa lata* had by far the highest relative abundance (91%) followed by two *Diatrypella* species (6.9% and 2.1%) previously reported in association with New Zealand *Vitis* and *Nothofagus* respectively [10, 27]. Rare Diatrypaceae sequences corresponded to *Eutypella* and *Peroneutypa* species (Table 1).

#### Family Sporocadaceae

Sporocadaceae accounted for 0.8% of total reads. The most abundant was classified as *S. vitis* (64%), a species which has not been recorded in New Zealand. Other OTUs belonging to Sporocadaceae included *Sporocadus rosigena*, a species isolated in this study, and a *Pestalotiopsis* sp. There were 11 low-abundance (rare) Sporocadaceae OTUs.

#### Other plant pathogens

*Dactylonectria*/*Ilyonectria* species, which have been associated with *Vitis* root diseases, were identified during screening against the TrunkDiseaseID database, but were rare. The wood-degrading basidiomycete *Schizophyllum commune* was detected, as was a second Schizophyllaceae species, *Chondrostereum purpureum* [28], during manual curation of the dataset. Other potential plant pathogens flagged using the FunGuild database included *Fusarium oxysporum* (1.14%), a *Peniophora* sp. (0.4%) and a *Neofabraea* sp. (0.38%) (Table 1). *Peniophora incarnata* is considered a saprophyte of grapevines [29] while *Neofabraea* species are often regarded as plant endophytes [30].

### Confirmation of OTU identity by specific PCR

Specific PCRs assays were designed to confirm the presence and identities of selected OTU sequences (Table 1). Focusing primarily on Diatrypaceae, Hymenochaetaceae and *Phaeoacremonium* fungi, we obtained high-quality DNA sequences from 19 samples. Re-amplifications were mostly targeted at moderately abundant OTUs, but a *Phaeoacremonium* DNA sequence was re-amplified from one sample containing only 0.02% of targeted reads. The remaining positive amplifications were from samples with at least 0.13% of reads. Three OTU sequences could not be confirmed by re-amplification; notably, reactions targeting a *Cryptovalsa ampelina* OTU (0.02–0.77%) returned poor-quality and/or *Vitis* sequences, while re-amplification (with both published and newly designed primers) targeting a rare *Lasiodiplodia theobromae* OTU returned DNA sequence with highest similarity to *D. mutila*.

### Fungal isolation from vines

To support the results from DNA metabarcoding, fungal isolation was performed from 40 vines at four vineyards. Fungal growth was observed from all vines, with 118 pure fungal cultures obtained. Based on colony growth forms and ITS DNA barcoding, 24 taxa were identified (Supplementary Table 1). The most frequently isolated species was *P. chlamydospora* (24 isolates), with *Epicoccum nigrum* (17) and a *Myrothecium* sp. (10) also common. Other cultured fungi from pathogen taxa included *S. rosigena* (7 isolates), *D. seriata* (6), *E. lata* (1), and an unnamed Diatrypaceae sp. (2) which has previously been detected in New Zealand [10].

### Disease measurements

Assessment of above-ground trunk disease symptoms found four vineyards with low rates of disease symptoms (0–2 diseased vines out of 45 vines) while seven vineyards had an intermediate number of symptomatic vines (6–13/45 vines) (Supplementary Table 2). One vineyard, initially included in this study but replanted due to disease pressure, had a large number of symptomatic vines (23/45). When averaged across samples, *E. lata* reads were more abundant in vineyards with intermediate than low levels of disease symptoms (Supplementary Fig. 2).

## Discussion

This study provides an unprecedented view of the trunk fungal microbiota across multiple vineyards in Marlborough, the largest grapevine-growing region in New Zealand. The aggregated nature of the DNA metabarcoding produced a vineyard-level view of fungal species presence, with several well-known trunk pathogens found to be widely distributed and of high relative abundance. We detected a similar range of basidiomycete and ascomycete fungi as found in prior culture-based studies of grapevine trunk tissues, including *Alternaria* spp., *Aureobasidium pullulans*, *Cladosporium* spp., *D. seriata*, *E. lata*, *E. nigrum* and *S. vitis* [5, 10, 26]. Detections of many fungal species were supported by parallel microbial isolations.

Distinguishing pathogens from other microbial species within complex metabarcoding datasets can be difficult, especially where multiple pathogen species may be present. Here, comparisons against a curated pathogens database [11] provided a simple means to identify globally recognised trunk pathogens. Further scrutiny of hits against the UNITE database produced an enlarged list of putative pathogens not detected by the TrunkDiseaseID approach.

### *Phaeomoniella chlamydospora* and *Eutypa lata*

A striking finding was the high relative abundance across almost all vineyards of the well-studied trunk pathogen *P. chlamydospora*. *Phaeomoniella chlamydospora* was also the most frequently isolated fungal species in culturing from a subset of the vineyards and has been widely detected in other studies across New Zealand [10]. International culture-dependent surveys have shown that *P. chlamydospora* is common in mature grapevines [26, 31].These studies support the interpretation that *P. chlamydospora* is genuinely abundant in the Marlborough vines, and this metabarcoding result was not due to PCR bias.

Despite the high levels of *P. chlamydospora*, esca symptoms were not seen in the vineyards. *Phaeomoniella chlamydospora* is considered a wilt pathogen, with pathogenicity attributed to xylem vessel clogging [32]. However, few recognisable pathogenicity genes in the *P. chlamydospora* genome also suggest an endophytic lifestyle [33]. *P. chlamydospora* has been isolated at equal abundance from apparently healthy plants as from esca symptomatic plants [26, 34]. Wood staining and vessel discoloration occur in *P. chlamydospora* pathology assays, but recapitulating the full range of esca symptoms is difficult [35]. *P. chlamydospora* may play a conditioning role, requiring other fungal species or changing environmental factors to induce disease. Esca is associated with a complex of fungi, especially *Phaeoacremonium minimum*, which is absent from New Zealand, but ‘tiger-stripe’ symptoms have been produced in pathology assays [36] with a combination of *P. chlamydospora* and *P. fraxinopennsylvanicum*, a species present in New Zealand. One vineyard where *P. chlamydospora* had high relative abundance and where there were few other candidate pathogens will be an important site for monitoring future disease development and vineyard longevity.

In addition to *P. chlamydospora*, *P. niveniae* was also widely detected in the Marlborough samples, albeit at lower relative abundances than for *P. chlamydospora*. The presence of this species was confirmed from multiple samples by specific PCR. Members of the Phaeomoniellales may be endophytes or occupy unknown ecological niches, with *P. niveniae* first detected as an endophyte from *Nivenia stokoei* (Iridaceae) [37]. Apart from a *Neophaeomoniella zymoides* specimen from a French grapevine [38], we are unaware of an association between Phaeomoniellales species (other than *P. chlamydospora*) and grapevines.

The second most abundant fungus in the dataset was another well-known GTD pathogen, *Eutypa lata*. This species has been detected in culture-dependent studies of mature grapevines in many countries [5, 26, 39] and is considered the main cause of eutypa-dieback in New Zealand [10]. In comparison with *P. chlamydospora*, the distribution pattern of *E. lata* was more punctuated: while abundant in some vineyards, it was nearly absent in others. Our analyses indicated that disease symptoms were most prevalent in vineyards with the highest average relative abundance of *E. lata*. Future studies of individual vines will be required to better understand the relationship between disease symptoms and trunk microbiology.

Beyond *P. chlamydospora* and *E. lata*, other well-known pathogens such as *D. seriata*, *D. mutila*, *S. vitis* and *Cadophora luteo-olivacea* were found to be widespread in Marlborough. *Diplodia seriata* was the least aggressive Botryosphaeriaceae species in Australasian pathogenicity tests [40, 41]. *Cadophora* species and *S. vitis* cause wood streaking in *V. vinifera*, but these species are not considered among the most aggressive of trunk pathogens in mature grapevines [42, 43]. These fungi may be of lower aggressiveness than other pathogens, or act in concert with other factors, to promote disease [44]; some GTD fungi are considered to behave as endophytes or latent pathogens until physiological perturbations induce disease development [45].

### Detection of new pathogens

Early detection of pathogen incursions may allow for interventions to prevent wider spread in the vineyard or growing region. Metabarcoding can be used to detect invading pathogens, although identifications may require corroborating evidence before action by growers is justified [46]. Here, specific PCR assays and Sanger sequencing confirmed the presence of many species first detected by metabarcoding. In particular, several Hymenochaetaceae were confirmed for the first time in New Zealand vineyards. In Europe, the white rotting stage of esca is associated with the hymenochaete *Fomitiporia mediterranea* [7]. In other jurisdictions [47, 48], endemic species of wood-degrading basidiomycetes fill this niche. The most abundant and widespread Hymenochaetaceae in Marlborough was *Inonotus nothofagi*, a species that infects native beech trees in New Zealand and Australia [27]. Although *I. nothofagi* is not known to infect other plant hosts in New Zealand (nzfungi2.landcareresearch.co.nz), beech growing in the adjacent Richmond ranges may provide an inoculum source for dispersal of *I. nothofagi* into Marlborough vineyards. Two additional detected Hymenochaetaceae species, one previously isolated in Marlborough [10] and a second related to *Fomitiporia austaliensis* were closely related to Australian fungi which have been implicated in grapevine white rot in Australia [49]. Further *Fomitiporella* and *Fomitiporia* species were confirmed by specific PCR. All of the Hymenochaetaceae displayed sequence differences to those from known species, supporting the presence of a region-specific collection of wood-degrading basidiomycetes in New Zealand vineyards. In contrast to *I. nothofagi*, the other detected Hymenochaetaceae were present at high relative abundance from only a few samples. Classical white rot symptoms have not been observed in New Zealand vineyards. Whether the newly detected Hymenochaetaceae species are degraders of dead wood, or play a role in disease onset, will require future single vine studies.

Most of the taxa confirmed by specific PCR were present at moderate relative abundance in the metabarcoding dataset, whereas new pathogen incursions might only be detected at much lower levels. Low relative abundance OTUs from two organisms of biosecurity interest to New Zealand, *Lasiodiplodia theobromae* [50], and *Cryptovalsa ampelina* [51], could not be confirmed by specific PCR. These metabarcoding identifications may represent sequencing anomalies, illustrating the difficulty of drawing conclusions based on rare OTUs. Resampling and individual vine analyses would be necessary before a response was justified.

This DNA metabarcoding has generated a wealth of information about the vineyard-level distribution of trunk fungi in the major grape production area of New Zealand. The resulting register of pathogenic fungi from Marlborough vineyards provides the basis for targeted single vine studies and future detections of pathogen incursions.

## Supplementary Information

**Additional file 1.**

## Data Availability

Data availability: Raw data are deposited in publicly available National Centre for Biotechnology Information Sequence Read Archive under BioProject accession number PRJNA658210. A requirement of this project was that individual vineyards would remain anonymous. Researchers can contact Bragato Research Institute (bri.co.nz), Blenheim, New Zealand to obtain further information about geographical coordinates of the vineyards studied for research purposes.
