# Supplementary material for: DNA metabarcoding reveals high relative abundance of trunk disease fungi in grapevines from Marlborough, New Zealand
Source: BMC Microbiol. 2022 May 10;22:126. doi: 10.1186/s12866-022-02520-2 (PMC9088082; doi:10.1186/s12866-022-02520-2)
Supplement: Supplementary file 1 — Additional file 1. [file 12866_2022_2520_MOESM1_ESM.docx]

**Supplementary Material**

Supplementary Table 1. Fungi isolated from Marlborough grapevine trunks (vineyards 17, 18, 20, 23). No. = total number of isolates grouped according to colony appearance. Taxonomic identity = putative identity based on best hits (>98% similarity) to the NCBI nt database by BLAST of ribosomal ITS sequence. Accession = GenBank accession numbers for a subset of isolates which were sequenced..

| **Taxonomic identity** | **No.** | **Accession** |
| --- | --- | --- |
| *Phaeomoniella chlamydospora* | 24 | MN206980-81 |
| *Epicoccum nigrum* | 17 | MN206989-93 |
| *Myrothecium* sp. | 10 | MN207004 |
| *Paraphaeosphaeria neglecta* | 8 | MN207003 |
| *Sporocadus rosigena* | 7 | MN206987 |
| *Aureobasidium pullulans* | 7 | MN206985 |
| *Diplodia seriata* | 6 | MN206982 |
| *Camarosporium brabeji* | 6 | MN206986 |
| *Alternaria alternata* | 5 | MN206984 |
| *Cladosporium cladosporioides* | 4 | MW142304 |
| *Lophiostoma* sp. | 3 | MN206983 |
| *Paraphoma chrysanthemicola* | 3 | MN207003 |
| *Pleosporales* sp. | 2 | MN206997 |
| *Diatrypaceae* sp. | 2 | MN206999 |
| *Eutypa lata* | 2 | MN209202 |
| *Coniochaeta* sp. | 2 | MN207002 |
| *Talaromyces verruculosus* | 2 | MN206988 |
| *Phaeococcomyces nigricans* | 1 | MN206994 |
| *Exophiala xenobiotica* | 1 | MN206995 |
| *Mycocalicium* sp. | 1 | MN206996 |
| *Pseudogymnoascus* sp. | 1 | MN206998 |
| *Vishniacozyma carnescens* | 1 | MN207000 |
| *Schizopora radula* | 1 | MN207001 |

Supplementary Table 2. Trunk disease symptoms. The five grapevines at each sampling locus (for a total of 45 vines/vineyard) were assessed for cankers, half-heads and canopy symptoms in the 2018-2019 season.

| **Vineyard_ID** | **Loci  with symptoms** | **Vines  with symptoms** |
| --- | --- | --- |
| 26 | 1 | 1 |
| 25 | 5 | 11 |
| 22 | 2 | 2 |
| 21 | 8 | 12 |
| 23 | 7 | 13 |
| 15 | 6 | 12 |
| 20 | 3 | 6 |
| 17 | 5 | 7 |
| 18 | 0 | 0 |
| 16 | 0 | 0 |
| 19 | 5 | 6 |


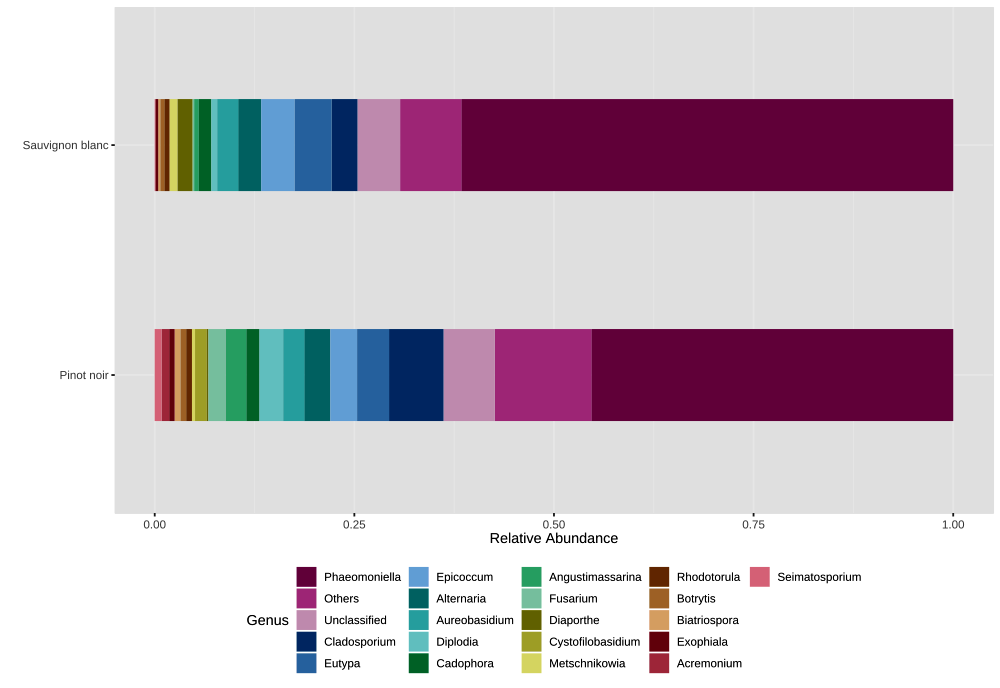


Supplementary Figure 1. Fungal abundance differences between varieties. Plots show the relative abundance of fungi in the two grape varieties, Sauvignon blanc and Pinot noir, averaged across the total dataset. Very low abundance species are grouped as ‘others’.


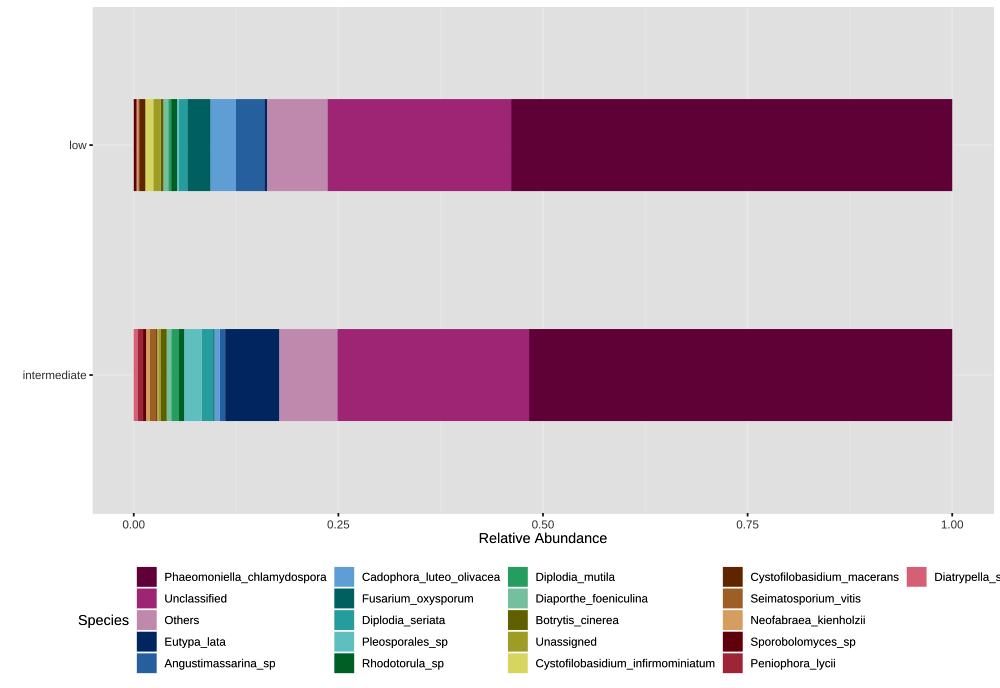


Supplementary Figure 2. Fungal abundance differences between vineyards with ‘low’ (0-2 symptomatic vines/vineyard) and ‘intermediate’ (6-13 symptomatic vines) disease symptoms. Plots show the relative abundance of fungal species in the vineyards, averaged across the total dataset. Very low abundance species are grouped as ‘others’.
